# Ethical Implications of Artificial Intelligence in Population Health and the Public’s Role in Its Governance: Perspectives From a Citizen and Expert Panel

**DOI:** 10.2196/44357

**Published:** 2023-04-27

**Authors:** Vincent Couture, Marie-Christine Roy, Emma Dez, Samuel Laperle, Jean-Christophe Bélisle-Pipon

**Affiliations:** 1 Faculty of Nursing Université Laval Québec, QC Canada; 2 School of Public Health Université de Montréal Montréal, QC Canada; 3 School of Research Sciences Po Paris Paris France; 4 Department of Linguistics Université du Québec à Montréal Montréal, QC Canada; 5 Faculty of Health Sciences Simon Fraser University Burnaby, BC Canada

**Keywords:** artificial intelligence, population health, citizen engagement, ethics, bioethics, digital app

## Abstract

**Background:**

Artificial intelligence (AI) systems are widely used in the health care sector. Mainly applied for individualized care, AI is increasingly aimed at population health. This raises important ethical considerations but also calls for responsible governance, considering that this will affect the population. However, the literature points to a lack of citizen participation in the governance of AI in health. Therefore, it is necessary to investigate the governance of the ethical and societal implications of AI in population health.

**Objective:**

This study aimed to explore the perspectives and attitudes of citizens and experts regarding the ethics of AI in population health, the engagement of citizens in AI governance, and the potential of a digital app to foster citizen engagement.

**Methods:**

We recruited a panel of 21 citizens and experts. Using a web-based survey, we explored their perspectives and attitudes on the ethical issues of AI in population health, the relative role of citizens and other actors in AI governance, and the ways in which citizens can be supported to participate in AI governance through a digital app. The responses of the participants were analyzed quantitatively and qualitatively.

**Results:**

According to the participants, AI is perceived to be already present in population health and its benefits are regarded positively, but there is a consensus that AI has substantial societal implications. The participants also showed a high level of agreement toward involving citizens into AI governance. They highlighted the aspects to be considered in the creation of a digital app to foster this involvement. They recognized the importance of creating an app that is both accessible and transparent.

**Conclusions:**

These results offer avenues for the development of a digital app to raise awareness, to survey, and to support citizens’ decision-making regarding the ethical, legal, and social issues of AI in population health.

## Introduction

Many researchers predict that the introduction of artificial intelligence (AI) systems powered by big data will have a huge impact on the health sector [1]. The authors anticipate that AI will enhance efficiency by automatizing routine tasks, respond to the shortage of health care professionals (HCPs), and offer more precise diagnosis and lower health care costs [2]. Although the benefits of AI are often presented from an individual perspective, particularly through the benefits of personalized medicine [3], AI does and can play a key role in improving the health of populations [4] because (1) the large-scale use of AI-based diagnostic or therapeutic tools would offer health benefits at the population level and (2) AI apps are being developed specifically for the population and public health. Examples include the use of AI-based technologies as part of public health interventions, such as surveillance programs aimed at predicting disease outbreaks [5]. AI has also been used for the automation of population screening in the cases of tuberculosis [6] and breast cancer [7].

In addition to these anticipated benefits, AI systems raise several ethical issues. The authors have pointed to the risks associated with the use of large data sets coming from specific groups of the population (eg, to train machine learning algorithms). It may not be possible to obtain individual informed consent to share personal health data in this context [8]. These technologies may threaten privacy [9] by making it possible to reidentify individuals [9] or collectivities [8]. Some authors also pointed out that the advent of AI in health care could replace the caring presence of health care providers and jeopardize the allocation of responsibilities for the interventions [1]. Another important group of issues is related to social justice. AI-based tools or programs could have been trained with incomplete or erroneous data that could exacerbate social prejudice toward more vulnerable groups [10]. Certain subgroups may be less represented in training data, leading to unequal performance of the AI system (eg, less efficient skin cancer detection in darker skin tones) [10,11]. Others have argued that focusing on individual characteristics (such as genetics, gender, race, and socioeconomic status), even in the context of prevention, could lead to neglecting social determinants of health [12]. Finally, AI-based apps also face the risk of exacerbating global injustices and create *data colonialism*, where “data act as the resource that is removed from its places of creation, changed, and then made into products that only marginally benefit those at its source” [13].

There is no consensus on the governance mechanism that should address these issues. The proposed strategies include letting private actors and corporations decide their own standards, whereas others favor public institutions to supervise the implantation of AI systems. One alternative to these top-down mechanisms is to mobilize all relevant stakeholders, including both experts and citizens [14]. Experts are important because of their knowledge of potential pitfalls and opportunities; their governing role through the publication of professional standards and guidelines; and their educational role, particularly in academia [15]. Citizens also play an important role in technological governance because AI systems may have impacts on individuals who do not directly use or have access to the technology or who may not have the choice to use it, for example, patients dependent on AI for their treatment [15]. In addition, citizen involvement in governing AI may help promote transparency and trust.

Corollary to the importance of engaging stakeholders in the governance process, few studies have tried to explore what form this involvement should entail [16]. To address this situation, we conducted a survey to explore the perspectives of a mixed panel of citizens, AI experts, scholars, and policy makers. The survey investigated three main themes: (1) the general perception of the panel regarding the ethics of AI in population health, (2) the engagement of citizens in AI governance, and (3) the potential of a digital app to foster citizen engagement. This allowed us to better understand the perspectives of experts and citizens regarding the ethical dimensions of AI in population health and the role of stakeholders, particularly citizens, in the governance of this type of AI use in the health sector.

## Methods

### Overview

From March to April 2022, we conducted a web-based survey with both quantitative and qualitative questions informed by a literature review on AI ethics in population health. The quantitative questions were close ended and 3-point and 7-point Likert scales. We used short-answer questions and open questions for the qualitative analysis. The survey ended with a 10-question sociodemographic questionnaire to characterize the sample. The survey was conducted in French using LimeSurvey (LimeSurvey GmbH), which is hosted on Université Laval servers. A link to the survey was sent directly to the participants.

### Recruitment and Sampling

Experts were recruited via professional email contact based on their expertise in AI, population health, or ethics and health law. Citizen recruitment was done through social media (Facebook and Twitter) as well as the mailing lists of Université Laval and the International Observatory on the Societal Impacts of AI and Digital Technology. Citizens were asked to complete a preapplication questionnaire and were selected by the research team to gather a diversity of participants in terms of gender, age, ethnicity, background, and level of knowledge on the topic.

### Data Analysis

For quantitative responses, we used Excel (Microsoft) for basic statistical analysis (means, medians, and SD) and SPSS (IBM Corp) to analyze the perceptions of respondents on different variables (level of expertise, gender, and age) and to generate *P* values. We used NVivo 12 (QSR International) to analyze qualitative responses using thematic analysis [17] to identify topics and arguments common to the respondents and more marginal ones that call for a broader discussion of our research object.

### Ethics Approval, Participation, and Informed Consent

The protocol and the survey were approved by the Comité d’éthique de la recherche de l’Université Laval, Université Laval’s Research Ethics Board (2020-124 R-1/10-05-2021). The study was considered to be of minimal risk for the participants. The participants signed an informed consent form before participating in the study.

## Results

### Overview

Participants shared their perspectives on three topics: (1) general considerations about AI in population health, (2) citizen engagement in AI governance, and (3) the potential of a digital app on the ethical challenges of AI in population health. The results are presented in the following sections, preceded by the sociodemographic characteristics of the sample.

### Participant Sociodemographic Characteristics

A total of 21 participants completed the survey (Table 1). The study population comprised 11 citizens and 10 experts, of which 62% (13/21) were women and 38% (8/21) were men. Of the respondents, 71% (15/21) were aged between 26 and 45 years, and 81% (17/21) of the respondents had graduated from a university (Table 1).

**Table 1:** Participant sociodemographic characteristics (N=21).

Characteristics		Values, n (%)
**Age (years)**		
	26-35	7 (33)
	36-45	8 (38)
	46-55	4 (20)
	56-65	1 (5)
	66-75	1 (5)
**Highest level of education completed**		
	High school diploma or equivalent	1 (5)
	Certificate or diploma from a college, a *Collège d'enseignement général et professionnel* (a postsecondary junior college in Québec), or any other nonuniversity institution	2 (10)
	University certificate or diploma lower than a bachelor’s degree	1 (5)
	Bachelor’s degree	4 (19)
	Master’s degree	5 (24)
	Doctorate	3 (14)
	Postdoctorate	5 (24)
**Gender**		
	Woman	13 (62)
	Man	8 (38)
**Level of expertise in the field of artificial intelligence**		
	No knowledge	1 (5)
	Novice	12 (57)
	Intermediate	5 (24)
	Advanced	1 (5)
	Expert	2 (10)
**Level of expertise in the field of population health**		
	No knowledge	2 (10)
	Novice	9 (43)
	Intermediate	5 (24)
	Advanced	3 (14)
	Expert	2 (10)
**Level of expertise in the field of ethics and law**		
	No knowledge	2 (10)
	Novice	5 (24)
	Intermediate	8 (38)
	Advanced	2 (10)
	Expert	4 (19)

### General Considerations About AI in Population Health

The data collected allowed us to draw a portrait of the participants’ general perceptions and attitudes toward AI in population health.

#### General Perception About AI Ethics in Population Health

**Figure 1:**
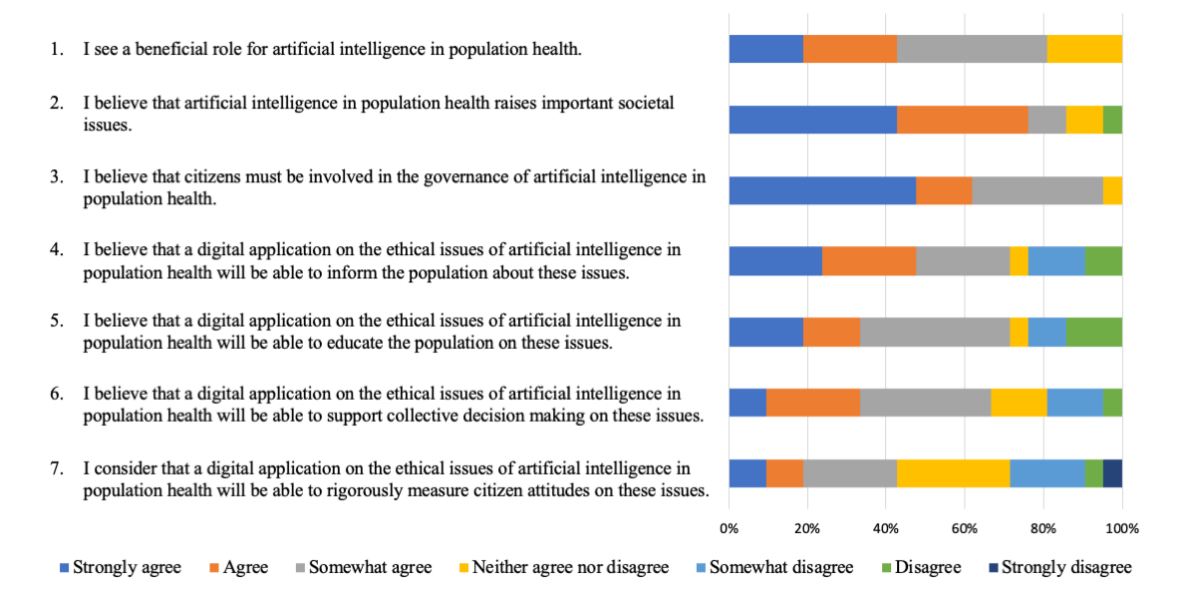
Participants’ answers to Likert scale questions.

Most participants (18/21, 86%) believed that AI was currently used in population health. Only 14% (3/21) of the participants indicated that they did not know. This last group is, on average, 10 years older than those who answered “yes.” The vast majority (17/21, 81%) strongly agreed, agreed, or somewhat agreed that AI plays a beneficial role in promoting the health of populations (Figure 1). The consensus was even stronger on the statement that AI in population health raises important societal and ethical issues (65.08%; Table 2).

In this context, participants were invited to describe the types of uses of AI they thought were used in population health. From their answers, 4 perceived uses of AI in health emerged (Textbox 1).

Participants were also surveyed on their relatives’ views on AI in population health. Participants were asked what they thought their relatives’ positive and negative opinions were on this topic. This question was intended to encourage respondents to think beyond their own opinions and not to probe the real perceptions of those around them. Some participants considered that the people around them were not aware of AI and its challenges; 1 participant explained this situation by their lack of interest (Textbox 2).

**Table 2:** Survey quantitative results.

Statements			Average overall agreement (N=21), mean (SD)
**General considerations about AI^a^ in population health**			
	“I consider that AI plays a beneficial role for the health of populations.”	1.43 (1.028)	
	“I consider that AI in population health raises significant societal issues.”	1.95 (1.322)	
**Citizen engagement in AI governance**			
	“I consider that citizens must be involved in the governance of AI in population health.”	2.05 (1.024)	
**Digital app on the ethical challenges of AI in population health**			
	“I consider that a digital application on the ethical issues of AI in population health will be able to inform the population about these issues.”	1.00 (1.871)	
	“I consider that a digital application on the ethical issues of AI in population health will be able to raise public awareness of these issues.”	0.71 (1.927)	
	“I consider that a digital application on the ethical issues of AI in population health will be able to support collective decision-making on these issues.”	0.81 (1.47)	
	“I consider that a digital application on the ethical issues of artificial intelligence in population health will be able to rigorously measure citizens’ attitudes toward these issues.”	0.29 (1.521)	

^a^AI: artificial intelligence.

**Perceived uses of artificial intelligence in population health.**

**Uses in population health**

- Public health response
- Population health
- Epidemiology and surveillance
- Prevention of diseases and health needs
- Decision-making (modeling, event management, and resources)

**Uses affecting individual health**

- Artificial intelligence–assisted surgery device
- Diagnosis, imaging, and detection
- Follow-up of chronic diseases

**Uses affecting the organization of health care**

- Patient support
- Making appointments
- Facial recognition
- Triage in emergencies
- Telemedicine

**Uses related to health research**

- Pharmaceutical
- Virology
- Data analysis

**Perceptions of participants’ relatives on artificial intelligence in population health.**

**Positive opinions of artificial intelligence in health**

- Usefulness for care
- Usefulness for health system management
- Usefulness for decision-making
- Usefulness for research

**Negative opinions about artificial intelligence in health**

- Bias and discrimination
- Invasion of privacy and surveillance
- Ecologic burden
- Impact on care
- Technophobia
- Not for the common good

#### Societal Challenges

The subsequent questions focused on the societal challenges posed by AI. For participants, AI in population health raised four types of societal challenges: (1) general challenges (comprising existential and technical questions); (2) social justice matters (issues about social inequalities, environment, and information); (3) risk of transformation of the health sector (for the patient and for health care workers); and (4) specific considerations for patients regarding privacy, autonomy, and the protection of their interests.

Some of the participants’ answers raised broad questions. Participants mentioned that AI could not perfectly reflect reality. They questioned the competence of an algorithm to make decisions pertaining to living organisms and raised the possibility for the algorithms to misidentify diseases, especially rare or orphan diseases. They emphasized that AI cannot replace human judgment. Some respondents mentioned technical issues related to the quality of data and algorithms. One participant mentioned that AI models have a failure mode that may make them difficult to deploy in new circumstances (eg, settings and data sets) and that data labeling and cleaning are very fastidious for researchers*.* More generally, participants reported that AI can raise questions about its transparency and explainability and that AI validation requires a very rigorous process. On another note, 1 participant emphasized that AI could represent an existential challenge for humanity.

Among the societal challenges reported, 3 types of preoccupations regarding social justice were identified: social inequalities, environmental issues, and disinformation. First, the participants were concerned about potential biases in the design and the use of AI, which could lead to increased social inequality. For instance, they referred to the risks that AI exacerbates unequal access to health care, contributes to the discrimination of subpopulations, and enhances social inequalities as well as economic equity issues. Some participants underlined the digital divide (disparity in access to technologies) and the issues of fairness and diversity of data. Second, some participants targeted a more specific point about social justice, namely, environmental issues. Indeed, they made references to the ecological burden and greenhouse gas emissions because of the power demand for overarchiving and electronic medical devices. Third, 1 participant mentioned concerns about access to quality information while AI contributes to the spread of disinformation on social media.

Another societal issue raised by some respondents is related to the transformation of the health sector. Participants mentioned the risk that the introduction of AI could affect the “access to healthcare and caregivers.” Another response highlighted the participation of such technologies in the “commercialization of healthcare.” From the perspective of HCPs, respondents noted that “AI encourages the bureaucratization of work” and that the technology will contribute to the “transformation of healthcare professions.”

The last group of societal challenges pertained directly to patient health. A total of 3 considerations emerged. First, the participants were concerned about the protection of privacy and the risks of a breach of confidentiality. Some responses emphasized that the use of AI in health makes traditional deidentification procedures insufficient and may lead to the use of data by private actors. Second, some respondents mentioned issues related to the respect of the patient’s autonomy; in other words, they highlighted a possible disempowerment regarding an individual’s self-care. Third, one answer underlined that the use of AI in population health can serve interests other than patient well-being.

### Citizen Engagement in AI Governance

Participants were surveyed on their attitudes toward citizen involvement in AI governance (Figure 1). This part did not focus on the health sector and took a more general policy perspective.

#### The Importance of a Citizen Perspective

Except for 1 person who neither agreed nor disagreed, there seemed to be consensus that citizens must be involved in the governance of AI in population health (Figure 1). When asked about the importance of citizen perspectives on the governance of AI in population health compared with that of other stakeholders, participants overwhelmingly indicated that the citizen perspective is of equal importance to others (Table 3). However, participants tended to consider the perspectives of ethicists, lawyers, and population health experts more important than those of citizens. AI developers’ perspectives were considered somewhat equal to those of citizens. Politicians and public policy makers were the only actors that were considered by respondents to be somewhat less important than citizens (Table 3).

Positive correlations were found between the importance of citizen involvement in AI governance and that of other stakeholders (health AI specialists, AI developers, ethicists, and lawyers). Finally, we noted a correlation between respondents who thought citizen perspectives were paramount and an agreement that societal issues were important.

**Table 3:** Importance of citizen perspective in comparison with other stakeholders (N=21).

Statements	More important, n (%)	Equally important, n (%)	Less important, n (%)
“I consider that the contribution of citizen perspectives in the governance of AI in population health is as important as the contribution of politicians and public decision makers.”	5 (24)	15 (71)	1 (5)
“I consider that the contribution of citizen perspectives in the governance of AI in population health is as important as the contribution of AI developers.”	5 (24)	10 (48)	6 (29)
“I consider that the contribution of citizen perspectives in the governance of AI in population health is as important as the contribution of ethicists and jurists.”	1 (5)	12 (57)	8 (38)
“I consider that the contribution of citizen perspectives in the governance of AI in population health is as important as the contribution of population health specialists.”	0 (0)	13 (62)	8 (38)

#### Types of Citizen Involvement in AI Governance

The participants converged on the need to consider citizen perspectives in AI governance. Four types of citizen involvement were proposed, which can be classified into increasing levels of demand: (1) information, (2) consultation, (3) decision-making, and (4) involvement in AI design. These types of involvement can be considered as recommendations from the participants. In other words, participants expect that citizens play an engaged normative role in AI governance.

Nevertheless, the least engaging perspective is to expect citizens to have the right to be informed about AI governance for a better understanding of its issues. Indeed, participants’ answers mentioned the need for transparency. According to them, informed citizens would have a better decision-making capacity in AI governance and lead to better health outcomes. One member of the panel mentioned the necessity of collecting data on more vulnerable populations to make sound decisions regarding population health. A total of 3 participants wrote about the necessity of keeping the population informed about scientific news and discoveries in a transparent manner. For 1 participant, more general information on good health habits was a prerequisite for participation in the debate. Thus, informing citizens appears to be important in the governance of AI.

Perhaps more engaging was the posture that expects citizens to be consulted. Consultation refers to the importance of free speech and active participation in, for instance, ethics committees and focus groups. Some participants also specified what needs to be discussed in these consultations: finding a balance between specialists’ and citizens’ perspectives; defining the fields of acceptable uses of the technologies; determining the risks and acceptable error thresholds; and verbalizing expectations, beliefs, and fears regarding the use of AI in population health.

An even more demanding posture was to expect citizens to be involved in decision-making. According to the respondents, citizens have a role to play regarding decisions on the use of citizen data, policy development, and public investments. Some answers expressed that citizens could contribute to making humans accountable for the consequences of the use of AI and to establish a decision-making hierarchy to limit the impacts and consequences related to the integration of AI in society.

The most demanding posture was that of respondents, indicating that citizens should be involved in the very conception of AI systems and participate in the research and development process of AI systems.

### Digital App on the Ethical Challenges of AI in Population Health

The final group of questions pertained to the development of a digital app to foster citizen involvement in AI ethics. Two dimensions were explored: (1) the relevance of a digital app and (2) how such an app could be implemented.

#### Perspectives on the Significance and Relevance of a Citizen Engagement Digital App

Participants were surveyed about the potential impacts of a digital app on raising awareness and surveying citizens on the ethical issues of AI in population health (Figure 1; Table 2). The goal was to estimate whether a digital app would be able to contribute to four variables: (1) to inform, (2) to raise public awareness, (3) to rigorously measure citizen attitudes on the issues of AI, and (4) to support collective decision-making.

The results showed that the participants’ average agreement regarding the 4 variables lies between “somewhat agree” and “neither agree nor disagree.” For the respondents, it is more likely that a digital app will be able to inform the population about these issues, raise awareness, support collective decision-making, and measure citizens’ attitudes on these issues. Participants were more ambivalent about the capacity of an app to rigorously measure citizens’ attitudes than the other variables.

In parallel, we found four positive correlations between the variables: (1) “information” and “raising awareness,” (2) “information” and “support collective decision-making,” (3) “raising awareness” and “support collective decision-making,” and (4) “support collective decision-making” and “rigorously measure citizen attitudes.”

Looking at participants’ feedback on the survey, it is possible that some were confused about the difference between “information” and “raising awareness,” which can alter some of these correlations. Moreover, it was noticed that the participants who did not know whether AI is currently used in population health agreed more than the others with the statement that a digital app will be able to rigorously measure citizens’ attitudes on ethical issues.

#### Implementation of a Citizen Engagement Digital App

We surveyed participants on the barriers and key performance indicators (KPIs) of a citizen engagement digital app. By KPIs, we meant components measuring the effectiveness, feasibility, and practicality of such a tool to assess its potential for implementation. The participants identified 6 barriers and 5 KPIs. Barriers and KPIs were addressed by 2 different questions, but the answers echo each other. Of the 6 obstacles, 4 can be associated with the 5 success indicators, which can be presented as responses to the obstacles (Table 4).

**Table 4:** Perspectives on the idea of using a digital app for citizen involvement.

Barriers to the app	Associated key performance indicators
Accessibility	Accessibility Clarity
Question of its usefulness	Participation rate
Question of interest and participation from the stakeholders	Impact of the tool on decision makers
Transparency	Transparency
Financing	—^a^
Difficulty to generalize	—

^a^Not available.

The first barrier pertains to the accessibility of the project. It was found that only people already interested in the topic would use the app. Some answers converged into design challenges and the need to be accessible to all citizens, for instance, with different communication modalities and accessible vocabulary. In this regard, 2 of the 5 KPIs can be associated with the first barrier: the app’s “accessibility” and “clarity.” A simple and easy-to-use tool whose functions and limits are clear would determine the success of a generic app.

The second barrier relates to the usefulness of the tool. In some cases, this type of digital apparatus was considered inadequate. The third KPI, which is the “participation rate,” can be linked to this barrier. For participants, the success of a digital app would be defined by the number of users and its representativeness of the population, by the commitment capacity produced (consultation, type and number of discussions generated, etc), and by the type of knowledge users targeted by the app’s results (visibility and use by relevant organizations).

The third challenge was to spark the interest and to stir up the participation of citizens and decision makers. A lack of citizen participation and of collaboration between the relevant actors would undermine the app, especially because, for participants, there was a need to convince skeptics of AI and to ensure the inclusion of marginalized populations. This barrier can be associated with the fourth KPI: the “impact of the tool on decision makers.” Some answers asserted that the app could change practices and be relevant for decision-making.

The fourth barrier questioned the integrity and transparency of the app. It was emphasized that the tool could be the result of a researcher confirmation bias and could encounter difficulties in considering the experiences of users. However, transparency can also be a KPI for the app, depending on its transparency in its design and on ethics issues and decisions.

Regarding the last 2 barriers, participants identified the risks of conflicts of interest in the conception team, raising questions about the funding of such a project. Finally, participants raised the question of the ability to generalize the app’s results because of the narrowness of its scope.

## Discussion

### Principal Findings

This study offers insight into public perspectives on 3 topics: AI ethics in population health, the role of citizens in the governance of technology, and the capacities of a digital app to mobilize citizen participation. Overall, the literature on public perspectives on AI, ethics, and population health is limited, especially from the perspective of citizens. Morgenstern et al [16] conducted a rare empirical study on the perspective of experts in population health. Most empirical studies have focused on stakeholders’ perspectives on specific AI systems use for clinical purposes [18]. Although it is not the focus of this research, some connections between the literature and this study can be made.

### Public Attitudes About AI, Ethics, and Population Health

#### Overview

Looking at the results in more detail, the survey showed that participants were aware of the use of AI in population health, which was considered mostly beneficial, although it raised several ethical issues. Following Scott et al [18], it is important to note that positive attitudes do not necessarily result in the adoption of AI systems and negative attitudes in resistance toward AI. These attitudes simply reflect the predisposition of the sample toward AI uses in population health. The participants’ perspective that AI plays a beneficial role in population health echoes the literature that identifies enthusiasm about AI in medicine from patients [19] and population health experts [16]. In general, the literature reveals that patients have a more positive attitude toward AI in health care than other stakeholders [18]. Furthermore, it seems that the positive general attitude of patients about the beneficial role of AI is reinforced by the trust in clinician oversight [18]. From the perspective of HCPs, AI has a useful role in the health sector, although its benefits are mitigated by its potential pitfalls [20]. In this study, these pitfalls were associated with 4 major challenges.

#### General Challenges

The first group related to more general issues of AI in terms of its capacity to radically transform something fundamental about humanity and, at the same time, its incapacity to fully grasp reality. The participants questioned the decision-making capacity of AI algorithms and their consequences for human beings. For instance, they highlighted the possibility of a program to make a medical error such as misidentifying a disease. Khullar et al [21] found that different stakeholders have different attitudes in this regard, as the public is more likely than physicians to consider medical errors as a major problem. However, as Khullar et al [21] mentioned, “these issues have not been examined as they relate to AI.” These general results gain more significance in relation to the following 3 issues: social justice issues, issues related to the transformation of the health sector, and issues directly affecting the patient.

#### Social Considerations

Participants were concerned about the capacity of AI to exacerbate social inequalities by fostering the digital divide, discrimination, and economic equity issues. The literature supports these findings. Richardson et al [19] argued that patients were preoccupied with rising health care costs because of advanced technology. They were also worried about the impact of AI tools on social protection and how it could result in discrimination [19]. The participants also reported being concerned about misinformation on social networks, which seems to be a new contribution to the literature. This can be explained by the context of the study, as the participants completed the questionnaire in 2021 during the COVID-19 pandemic. At the time, the issue of fake news in the field of health (among other fields) on the internet was widespread in the media and in the daily lives of the participants. The results related to the environmental cost of infrastructure-sustaining AI are another emerging topic. Although well-known and discussed in certain circles [22], they have not yet been fully recognized by empirical bioethics studies of AI.

#### Transformation of the Health Sector

Some participants agreed that AI would transform or replace HCPs. This is in line with the literature, which shows that the public is more likely to have pessimistic attitudes toward AI in health compared with HCPs [20,21]. This can explain our results because a large proportion of the sample was composed of people without specific expertise in population health—the health care domain that was surveyed. Participants in the study were concerned that AI could modify the access to care for patients through processes such as the commercialization of health care. Similarly, the literature shows that patients surveyed are usually worried about the future of care because of the increasing dependency of health care on digital tools [19], and they expect a replacement of humans by computers and robots [20]. In this context, patients are usually worried about the dehumanization of relations with HCPs and may manifest low levels of trust in AI [18,21]. In contrast, most of the workers in the study by Castagno and Khalifa [20] recognized a useful role for AI in health, and only a few of them were concerned that they would lose their jobs to algorithms. The authors found that there would be some resistance to change from HCPs, correlated with a lack of understanding of AI [20]. Thus, it seems that there are 2 groups of positions on the transformation of health care: predisposed patients and resistant HCPs. In addition, the literature shows a certain optimism about reduced workload and bureaucratization [21], matching the claim of our participants, according to which AI could encourage a positive transformation in the administrative area.

Participants did not raise the issue of liability for medical errors made by AIs, although this is an important topic in the literature [19,21]. A study by Khullar et al [21] showed that the public believes that the physician should be held responsible, whereas health care specialists believe that the liability is incumbent on third parties, such as vendors and organizations. In this debate, it seems that academics and patients draw a consensus: “the physician must be at the centre of medical decision-making to preserve patient safety” [19].

#### The Human Dimension of Health Care

The participants addressed direct considerations for patients. For them, AI in population health may lead to a certain preoccupation with privacy, security, and confidentiality. Some respondents were concerned about the use or ownership of confidential data (such as personal health information) by private stakeholders. These results are supported by the literature on security matter [19-21]. Health care staff, specialists, and patients agreed that privacy issues may emerge from the propagation of AI in health care [20]. Regulations are perceived as a necessity to prevent such threats [19]. Patients feel that HCPs should be transparent in the event of an error by an AI system, which is a duty related to their full responsibility [19]. In addition, 1 participant has suggested that the use of AI in population health may serve other interests than patients’ well-being, which is supported by the literature that converges toward a concern for commercial motives [21].

According to the participants in our study, AI cannot replace the judgment of HCPs, and the human dimension of care is fundamental. The results from another empirical study follow the idea that AI systems should not be autonomous; decisions and monitoring should remain the tasks of a human being. Moreover, the patient must be involved in the decision-making process independent of the involvement of AI in care [19]. It seems that citizens would react negatively if AI were used alone in health care, without staff assistance, indicating a lack of trustworthiness despite the acknowledged benefits.

Participants also reacted to the possible disempowerment of the population regarding their own health. This resonates with the literature on AI’s potential to damage patient autonomy [23]. It is possible to imagine the alienation of human beings with their health, as their responsibility toward their body would be entrusted to an algorithm. Participants talked about a loss of responsibility toward themselves, as AI becomes responsible for a person’s health. Therefore, according to the participants, a double anthropogenic effect may be observed if the transformation of the health sector by the implementation of AI is not done cautiously: a loss of human connection between the patient and HCPs and a loss of connection with oneself.

### Citizen Engagement in AI Governance

#### Overview

A key insight from the survey was the consensus on the importance of citizen involvement in AI governance. This may seem relatively minor to some; this type of questioning is part of a long discussion on the place that stakeholders should have in AI governance and ethics [14]. For instance, Stahl [15] stresses the idea that stakeholder engagement is needed to steer positive change and to ensure more democratic and human-centric uses of AI. People-oriented AI uses and governance are key to ensuring the sound and trustworthy uses of AI. This entails ensuring that adequate democratic control is put in place to inform and contribute to the governance and political deliberation regarding AI [24].

Obviously, asking such a question to a panel composed of citizens and experts involved in ethics, technological governance, or population health calls for a favorable opinion regarding citizen input. However, even for some advocates of a greater democratization of citizen presence in AI governance, it is not always clear how to get this level of buy-in to the idea, let alone a consensus [14]. For example, Himmelreich [25] argued that democratic endeavors to AI ethics and governance are not only a matter of involving citizens (or as many citizens as possible). It also entails that they are meaningfully engaged in ways that lead to informing and supporting real (and not speculative) decision-making that may result in veritable changes. However, these types of reservations (both regarding the end of citizen involvement and the means to achieve it) are much less likely to arise in the health sector. The health sector and a fortiori in the population health sector call for significant citizen engagement and the social acceptability of health policies, interventions, and products. Health deserves specific consideration, as it is widely conceived as a fundamental value that implies distinguishing technological governance in health in a different way than most other social sectors [26]. As a result, many movements call for the citizen perspective to be included in the design of health governance. This is what some call the “engagement turn in health governance and ethics” or the “nothing about us without us*”* movement [27].

It is interesting to note the different levels of citizen involvement proposed by the participants: (1) information, (2) consultation, (3) decision-making, and (4) involvement in AI design. This indicates a progressive level of demand for the extent to which citizens are engaged in governance. Interestingly these 4 different levels suggested by respondents echo the categories of the Spectrum of Public Participation of the International Association for Public Participation [28]. The Spectrum of Public Participation of the International Association for Public Participation divides the last category of public engagement, which is “decision-making” into “collaborate” and “empower.” According to the spectrum, the higher form of public engagement is “to place the final decision-making in the hands of the public.” This avenue should be explored in the future to determine how it could be implemented in the context of AI in population health.

#### Implication of Education and Trustworthiness to Guarantee Citizen Involvement

A normative implication of this study is the need to educate the public. When citizens were asked if they could think of AI technologies in population health, many named general types of uses not necessarily related to population health (Textbox 1). Similarly, in a study on patients’ and HCPs’ perspectives on AI in health, Castagno and Khalifa [20] found that both groups had little knowledge of the topic. A general need for education on AI and its uses in population health seems imperative and can become increasingly urgent depending on the ethical issues correlated. The main idea is to avoid growing skepticism about AI in population health [19] because of a lack of involvement due to poor understanding.

### Requirements for the Creation of a Digital App

The participants appeared to be relatively ambivalent about the use of a digital app to mobilize citizen engagement in the ethics of population health AI. As O’Connor et al [29] suggested, citizens’ engagement in a health-related app may be less straightforward than simply asking their general opinion. The authors pointed out that engaging with a digital app requires several prior steps. This is not sufficient to theoretically reflect on the potential use of an app. Concrete steps need to be implemented, such as providing support for its use and allowing users to actively try the app. In that sense, the engagement becomes the result of the process.

Furthermore, we observed that the barriers and KPIs identified by the participants echo each other, indicating that to be valid, an app should overcome these barriers. The issue of “accessibility” is particularly important in the field of digital tools and relates to the “digital divide,” also referred to by the participants (although not necessarily using that term). Indeed, these tools are not equally accessible to all segments of the population; age, level of education, level of digital literacy, and access to a sufficiently good internet network are factors that influence access [30]. As noted by the participants, unequal access to digital tools could lead to a lack of diversity and representativeness of data. However, accessibility (and clarity) issues can be considered in the tool’s development phase to incorporate features that circumvent these problems [31]. Moreover, access to the internet and a connected device is high in countries such as Canada, where our app aims to be deployed (nearly 9 out of 10 people in Canada have access to broadband internet [32]). The use of digital tools could even increase the representativeness of often underrepresented groups such as youth [31]. Greater accessibility to a digital tool should translate into increased participation or is at least a prerequisite for it. In turn, greater participation increases the ability to generalize the results [31], a barrier emphasized by participants. In addition, greater use of the app would increase its visibility and ability to be considered by decision makers. Therefore, accessibility (and clarity) is fundamental for mitigating barriers and addressing the KPIs identified by participants.

The participants also mentioned the importance of “transparency.” The involvement of citizens in the development of tools can increase its transparency [14]. Similarly, disclosure of the financial sources for the development of the tool can mitigate fears of conflicts of interest and increase public confidence in the app [33]. This last point is particularly important, given that private corporations have a large share in the development of AI-related tools [34].

To work on the issue of the tool’s “generalizability of results,” a great importance should be given to its methodological standards, including the choice of an appropriate methodology and the consideration of the tool’s inherent limitations. In addition, standards applicable to digital tools, such as data quality, should be taken into account [35].

Research on digital apps involving the public in AI ethics is still in its early stages. This echoes the work conducted in the emerging fields of design bioethics [31] and digital bioethics [35]. Schneider et al [35] posit that digital bioethics pushes forward the empirical turn of bioethics. According to Pavarini et al [31], design bioethics is a novel way of doing bioethics at the intersection of research, public education, and citizen education. Following the call of these authors, further studying the perceptions of stakeholders on how they should be involved appears to be essential.

### Limitations

The small sample size is the main limitation of this study. The study was limited to 21 participants, largely living or working in a French-speaking context. In addition, the citizen participants, recruited via social media and academic mailing lists, had a very high level of education—much higher than that of the general population; this represents a bias and a finding in itself, showing that people interested in AI in health are possibly more educated. However, the respondents’ varied profiles and the diversity of the themes surveyed allowed for a rich analysis of a topic that is still relatively unexplored empirically on both citizens’ and experts’ perspectives. There was no statistical hypothesis testing, and the quantitative results cannot be generalized because of the modest group of respondents. This study presents preliminary results that can be useful for the initial discussion and observations on the ethical issues of AI in population health and on citizen engagement in this area. This represents an emerging research area that should be further explored using larger and more diverse populations in a global context.

### Conclusions

According to the participants, AI is perceived to be already present in population health. Participants’ attitudes about AI in population health showed a positive general predisposition about the opportunities that AI can lead to, but without overconfidence. Participants agreed that AI raises significant societal implications, inter alia, regarding the security of the technology, social justice issues, and the risk of dehumanizing health care. The participants demonstrated a high level of agreement for involving citizen into AI governance. To make this possible, it would be necessary to transfer knowledge to the population and build trustworthiness in AI, regardless of the stakeholders. Finally, the respondents highlighted avenues for the use of a digital app to foster this involvement. A main task relates to creating an app that is accessible knowing that the topic of AI in population health is a complex one and, at the same time, recognizing that such digital app faces limits because of variable digital literacy in the population. Another important task is to make an app that is transparent. Trustworthiness appears to be an important value not only for the implementation of AI in population health but also in the design of an app dedicated to mobilizing citizens. These conclusions aim to guide the development of a digital app to raise awareness, to survey, and to support citizens’ decision-making regarding the ethical, legal, and social issues that AI raises in population health. These results are informative, particularly regarding the roles of citizens, and provide a better understanding of their importance in relation to other stakeholders. Future research will lead us to put into practice the insights from the panel and apply them to the development of a concrete citizen-oriented app that can help respond to the expectations of concrete citizen engagement in AI governance.

## Abbreviations

AI: artificial intelligence
HCP: health care professional
KPI: key performance indicator
